# Assessment of *TLL1* variant and risk of hepatocellular carcinoma in Latin Americans and Europeans

**DOI:** 10.1016/j.aohep.2023.101181

**Published:** 2023-11-20

**Authors:** Siyu Fu, Dhamina Karim, Jhon Prieto, Domingo Balderramo, Javier Diaz Ferrer, Angelo Z. Mattos, Marco Arrese, Enrique Carrera, Jeffrey Oliveira, Jose D. Debes, Andre Boonstra

**Affiliations:** a Department of Gastroenterology and Hepatology, Erasmus MC University Medical Center, Rotterdam, The Netherlands; b School of Public Health, University of Minnesota, Minneapolis, MN, USA; c Centro de Enfermedades Hepaticas y Digestivas, Bogota, Distrito Capital de Bogota, Colombia; d Hospital Privado Universitario de Córdoba, Instituto Universitario de Ciencias Biomédicas de Córdoba, Cordoba, Argentina; e Facultad de Medicina, Universidad de San Martin de Porres, Lima, Perú; f Graduate Program in Medicine: Hepatology, Federal University of Health Sciences of Porto Alegre, Porto Alegre, Brazil; g Departamento de Gastroenterología, Facultad de Medicina, Pontificia Universidad Católica de Chile, Santiago, Chile; h Hospital Especialidades Eugenio Espejo, Universidad San Francisco de Quito, Quito, Ecuador; i Department of Medicine, University of Minnesota, Minneapolis, MN, USA

**Keywords:** *TLL1*, Hepatocellular carcinoma, Latin America, Liver, Cancer

## Abstract

**Introduction and Objectives:**

Tolloid like protein 1 (*TLL1*) rs17047200 has been reported to be associated with HCC development and liver fibrosis. However, to our knowledge, no studies have been performed on Latin Americans and comparative differences between *TLL1* rs17047200 in HCC patients from Latin America and Europe are undefined.

**Materials and Methods:**

Cross-sectional analysis performed on Latin American and European individuals. We analyzed *TLL1* rs17047200 on DNA from 1194 individuals, including 420 patients with HCC (86.0 % cirrhotics) and 774 without HCC (65.9 % cirrhotics).

**Results:**

*TLL1* rs17047200 genotype AT/TT was not associated with HCC development in Latin Americans (OR: 0.699, 95 %CI 0.456–1.072, p = 0.101) or Europeans (OR: 0.736, 95 %CI 0.447–1.211, p = 0.228). *TLL1* AT/TT was not correlated with fibrosis stages among metabolic dysfunction-associated steatotic liver disease (MASLD) patients from Latin America (OR: 0.975, 95 %CI 0.496–1.918, p = 0.941). Among Europeans, alcohol-related HCC had lower *TLL1* AT/TT frequencies than cirrhosis (18.3 % versus 42.3 %, OR: 0.273, 95 %CI 0.096–0.773, p = 0.015).

**Conclusions:**

We found no evidence that the *TLL1* rs17047200 AT/TT genotype is a risk factor for HCC development in Latin Americans or Europeans. A larger study integrating ethnic and etiology backgrounds is needed to determine the importance of the *TLL1* SNP in HCC development.

## 1. Introduction

Hepatocellular carcinoma (HCC) is the primary malignancy of the liver and occurs predominantly in individuals with underlying liver disease or cirrhosis [1]. HCC accounts for approximately 800,000 deaths annually, making it one of the most lethal cancers in adults [2,3]. Host genetics play an important role in predisposing individuals to the risk of fibrosis progression and HCC. In this regard, several single nucleotide polymorphisms (SNPs) have been reported to be associated with HCC development [4]. One SNP, rs17047200, is located in the Tolloid like 1 (*TLL1*) gene on chromosome 4 [5−7] and encodes for a metalloprotease.

Genome-wide association studies from Japan showed a strong association between the *TLL1* variant and HCV patients who later developed HCC after antiviral treatment [5,6]. However, studies performed in European [7] and Egyptian populations [8] did not show any association between *TLL1* polymorphisms and cirrhotic HCV patients who later developed HCC after viral treatment. In addition, a study from Japan showed advanced fibrosis in patients with metabolic dysfunction-associated steatotic liver disease (MASLD) associated with *TLL1* genotype AT/TT compared to AA [9]. However, these results were not confirmed in a cohort of Caucasian patients with MASLD [10]. These studies suggest a differential association of *TLL1* in HCC depending on the population.

To our knowledge, no studies have been performed to assess *TLL1* polymorphisms and the risk of HCC in Latin Americans, a population with a high incidence of both MASLD and HCV-related HCC [11,12]. In this study, we evaluated the risk association of the *TLL1* SNP in a well-defined Latin American cohort with HCC and compared it to a European cohort.

## 2. Materials and Methods

### 2.1. Selection criteria for patient inclusion

A cross-sectional study analysis was conducted using data from the ESCALON network [13,14], which is a European-Latin American network that evaluates clinical and genetic factors for discovering biomarkers in early diagnosis and treatment of hepatobiliary tumors (www.escalon.eu). HCC patients were diagnosed through radiological evidence in accordance with European and American [15,16]. Patients with sufficient information on liver disease etiology, tumor size, and fibrosis status were included. The exclusion of patients includes HCC recurrence, non-HCC liver metastases, mixed-type HCC, age < 18 years, and co-existing non-HCC malignancies.

### 2.2. Patient cohorts

This cohort included patients from Latin America (Argentina, Brazil, Chile, Colombia, Ecuador, and Peru) and Europe (The Netherlands) since 2019. Medical records and confirmatory imaging, pathology, and laboratory tests were used to identify the etiology, tumor stage, and fibrosis stage. Details on etiology, tumor stage, and other information can be found in Table 1.

Etiology assessment: Patients with HBV or HCV infection were diagnosed serologically. Patients with MASLD had either a diagnosis assigned by the managing hepatologists or evidence of hepatic steatosis by histopathology or ultrasound, without other liver damage triggers. Patients with alcoholic liver disease (ALD) had persistent steatohepatitis following an estimated daily ethanol intake of more than 40 g/day for men and more than 30 g/day for women for over 10 years, in the absence of other liver diseases. Other etiologies included alpha-1 antitrypsin deficiency, acquired immune deficiencies, autoimmune liver disease, hemochromatosis, primary biliary cholangitis, primary sclerosing cholangitis, Wilson’s disease, a documented clinical history of immunomodulatory drugs, pathologyproven Metavir F2-F3 fibrosis without risk factors, and monogenic syndromes. Patients with HBV or HCV infection were assigned to the viral group, and patients with MASLD or ALD were assigned to the non-viral group.

Tumor stage and fibrosis evaluation: For patients with cirrhotic HCC, the Barcelona Clinic Liver Cancer (BCLC) staging system was used [17]. The presence of severe fibrosis or cirrhosis was established by the managing hepatologists through pathology (Metavir ≥F3-F4) or liver transient elastography studies (> 12.0 kPa).

### 2.3. Serum collection

Serum was collected prospectively starting from 2019 and onwards specifically for HCC biomarker discovery and validation studies. A data monitor regularly checks all data. The control group was required to have a minimum follow-up of 24 months after biomarker assessment to confirm the absence of HCC. Serum samples from patients diagnosed with HCC were collected at the time of diagnosis. DNA was isolated from the peripheral blood of 1194 patients from Argentina, Peru, Chile, Colombia, Brazil, Ecuador, and the Netherlands (as a European control cohort).

### 2.4. TLL1 SNP

We analyzed the proportion of the rs17047200 variant located within the *TLL1* gene for individuals with and without HCC within the Latin American and European populations. The *TLL1* rs17047200 SNP was genotyped using TaqMan probe: TTTTGCCCACTTATGTCCATTTCAC [A/T] GTTCATTGACATCTATTTCTGAAGG (ThermoFisher, cat. no. 4351376). Genotyping was performed using StepOnePlus Real-Time PCR System (ThermoFisher) and a Custom TaqMan SNP Genotyping Assay (Applied Biosystems).

### 2.5. Statistics

Statistical analyses were performed by SPSS 28.0.1.0 (SPSS Inc., Chicago, IL, USA). Continuous variables were presented as medians, and categorical variables as percentages. The Mann−Whitney U test was used to test continuous variables, and chi-square and Fisher’s exact tests were used to test dichotomous variables. Logistic regression was used to examine the association between HCC and the *TLL1* variant. A two-tailed value of p < 0.05 was considered statistically significant.

### 2.6. Ethical statements

Written informed consent was obtained from each patient included in the study and the study protocol conforms to the ethical guidelines of the 1975 Declaration of Helsinki as reflected in a priori approval by the local and/or regional Ethics Committees of all centers.

## 3. Results

The study included 1,194 individuals, including 420 HCC (86 % cirrhotic) and 774 controls (65.9 % cirrhotic). As presented in Table 1, the Latin American cohort consisted of 218 individuals with HCC (90.4 % cirrhotic), 333 with cirrhosis without HCC, 99 with chronic viral hepatitis, and 53 healthy controls. The European cohort consisted of 202 individuals with HCC (81.2 % cirrhotic), 177 with cirrhosis without HCC, 106 with chronic viral hepatitis, and 6 healthy controls. The median age of HCC individuals was 68 years (IQR: 61 to 74) among Latin Americans and 68 years (IQR: 62 to 72) among Europeans. 59 % and 77 % of Latin American and European with HCC were males, respectively. The most common cause of HCC in the Latin American cohort was MASLD (48 %), while in the European cohort it was ALD (35 %). Detailed characteristics of the study populations by HCC status with statistical analyses are summarized in Table 1.

In the Latin American cohort, the proportion of the *TLL1* pathogenic variant AT/TT among HCC patients was lower than among patients with cirrhosis (18.8 % versus 25.0 %), but no statistical difference was found (p = 0.093, Table 1). Similarly, European patients with HCC had a lower proportion of *TLL1* AT/TT than those with cirrhosis without HCC (23.3 % versus 28.2 %, p = 0.268, Table 1). In addition, no significant differences were observed in the comparison of HCC patients from Latin America and Europe (18.8 % versus 23.3 %, p = 0.262). When adjusted for gender, age, and etiology (MASLD or ALD), *TLL1* AT/TT was found not to be a risk factor for the development of HCC in Latin Americans when compared to patients with cirrhosis (OR: 0.699, 95 %CI 0.456–1.072, p = 0.101, Fig. 1, Table 2). A similar finding was observed when comparing HCC versus cirrosis in the European cohort (OR: 0.736, 95 %CI 0.447–1.211, p = 0.228) (Fig. 1, Table 2). Next, we analyzed the association of *TLL1* AT/TT in a Latin American cohort with self-reported Latin American ancestry (n = 185) and found that the SNP did not represent a risk factor for HCC (OR: 0.643, 95 %CI 0.285–1.451, p = 0.288; adjusted OR: 0.551, 95 %CI 0.224–1.353, p = 0.193) compared to cirrhosis.

The minor allele frequency (MAF) of *TLL1* rs17047200 A>T in the public database (dbSNP, www.ncbi.nlm.nih.gov/snp/) was 11.3 % (sample size 610) and 13.1 % (sample size 16,444) for Latin Americans and Europeans, respectively. In our study cohort, the minor allele frequencies were similar for patients with HCC (Latin America 10.0 %, Europe 15.4 %) or cirrhosis (Latin America 13.5 %, Europe 17.2 %) compared to the dbSNP database.

Next, we performed sub-group analysis on underlying liver disease (MASLD, ALD, viral hepatitis). We found that the *TLL1* genotype AT/TT is not associated with viral- or MASLD-related HCC compared to cirrhosis in both the Latin American or the European cohort (p > 0.05) (Table 3). Interestingly, in the European cohort, patients with ALD-related HCC (13 out of 71) exhibited a lower frequency of *TLL1* AT/TT genotypes (18.3 % versus 42.3 %, p = 0.015) than those with cirrhosis (11 out of 26, OR: 0.273, 95 %CI 0.096–0.773, p = 0.015, Table 3), which was predominantly due to a lower frequency of the AT genotype. In contrast to Europeans, no difference was found among Latin American ALD-related HCC patients (22.5 %, 9 out of 40) compared to cirrhosis (31.1 %, 19 out of 61, p = 0.342) as assessed by chi-square test.

Studies have reported that the *TLL1* genotype AT/TT is associated with fibrosis stages in MASLD patients from Japan [9]. To examine this, we assessed the proportion of the *TLL1* pathogenic variant (AT/TT) among MASLD individuals without cirrhosis (n = 78) and with cirrhosis (n = 174) in Latin Americans. No statistical difference was observed for the proportion of the *TLL1* genotype AT/TT between MASLD-hepatitis (21.8 %, 17 out of 78) and MASLD-cirrhosis (24.7 %, 43 out of 174, p = 0.615), suggesting that in Latin America the *TLL1* genotype AT/TT was not associated with fibrosis stages in MASLD patients (adjusted OR: 0.975, 95 %CI 0.496−1.918, p = 0.941).

## 4. Discussion

This is the first report on the association between the genetic variant *TLL1* rs17047200 and HCC in a cohort of Latin American patients compared to Europeans. We found that a *TLL1* polymorphism is not associated with HCC development in either population. Also, the *TLL1* AT/TT genotype does not appear to be associated with MASLD- or viral-related HCC development as well as the fibrosis stages of MASLD. Although two studies from Japan found an association between *TLL1* rs17047200 and HCC development after eradication of HCV infection [5,6], two longitudinal studies from Europe [7] and Egypt [8] suggested that the *TLL1* variant is not a predictive biomarker for HCV patients who later developed HCC after antiviral treatment. In our cohorts, we were unable to analyze this due to a limited group size of HCV-infected patients. Also, different from the longitudinal Japanese studies, we conducted a cross-sectional study since we collected serum from HCC patients the first time of diagnosis. It should be noted that cirrhotic controls were followed for a minimum of 24 months. However, in the analysis of HCC linked to the viral infection group we did not observe an association, which may relate to different population characteristics. Ethnic background may be important, but no difference was found in the Latin American cohort when we divided patients according to self-reported ancestry. Our study did not count with enough individuals of reported Asian ancestry to perform a sub-group analysis in this group. It should be noted that self-reported ethnicity is not as accurate as patient’s genetic information. Although one Japanese study found that the combination of *PNPLA3* and *TLL1* polymorphisms can predict the advanced fibrosis stage in MASLD patients [9], no association between the *TLL1* genotype itself and fibrosis stages of MASLD patients was observed in our Latin American cohort. In contrast, in the European cohort, ALD-related HCC patients had a lower frequency of *TLL1* AT/TT genotype than cirrhosis patients, which was not observed for the Latin American cohort.

## 5. Conclusions

For the first time, this study revealed a negative association between *TLL1* rs17047200 and the occurrence of HCC in patients from Latin America. Our study highlights the importance of addressing specific ethnic cohorts and etiology when assessing the genetic risk of HCC. A larger study confirming these findings and integrating specific ethnic and etiology backgrounds within groups is warranted.

## Figures and Tables

**Fig. 1.**
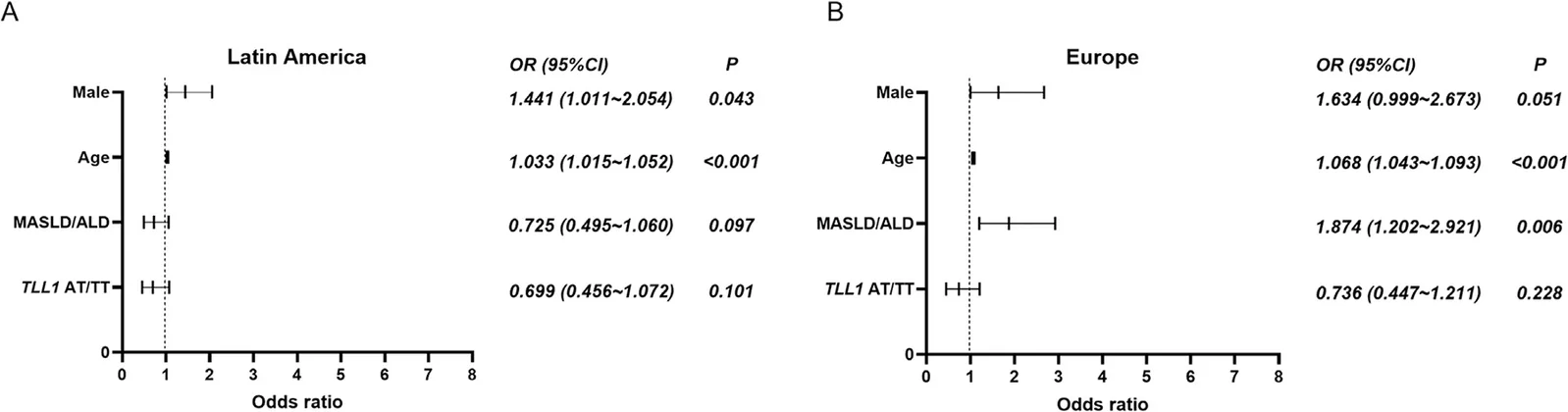
Logistic regression analysis was performed on HCC patients and cirrhotic non-HCC patients from Latin America and Europe. (A) In Latin America, age and male are risk factors for HCC development. (B) In Europe, age and etiology (MASLD and ALD) are risk factors for HCC. *TLL1* AT/TT is not a risk factor for HCC development in either population. References for *TLL1* AT/TT, etiology (MASLD and ALD), and male are *TLL1* genotype AA, other etiologies, and female, respectively. Abbreviations: ALD, alcoholic liver disease; HCC, hepatocellular carcinoma; MASLD, metabolic dysfunction-associated steatotic liver disease; TLL1, Tolloid like 1; OR, odds ratio.

**Table 1:** Patient’s information of the Latin American and European cohort

	Latin America				Europe				
Variable	HCC (n = 218)	Cirrhosis (n = 333)	Hepatitis (n = 99)	Healthy controls (n = 53)	HCC (n = 202)	Cirrhosis (n = 177)	Hepatitis (n = 106)	Healthy controls (n = 6)	P-value

Age, median (IQR)	68 (61–74)	64 (58–70)	59 (50–64)	47 (32–65)	68 (62–72)	60 (52–67)	53 (42–62)	50 (32–54)	0.718
Male, n (%)	130 (59.6%)	175 (52.6 %)	37 (37.3 %)	8 (15.1 %)	156 (77.2 %)	120 (67.8%)	61 (57.5 %)	5 (83.3 %)	<0.001
Cirrhosis, n (%)	197 (90.4%)	333 (100 %)	NA	NA	164 (81.2 %)	177 (100%)	NA	NA	0.007
Etiology, n (%)									
HBV	8 (3.7 %)	7 (2.1 %)	14 (14.1 %)	NA	19 (9.4%)	12 (6.8 %)	61 (57.5 %)	NA	0.017
HCV	23 (10.6%)	29 (8.7 %)	5 (5.1 %)	NA	23 (11.4%)	59 (33.3 %)	7 (6.6 %)	NA	0.784
MASLD	105 (48.2%)	174 (52.3 %)	78 (78.8 %)	NA	40 (19.8 %)	35 (19.8 %)	15 (14.2 %)	NA	<0.001
ALD	40 (18.3 %)	61 (18.3 %)	1 (1.0%)	NA	71 (35.1 %)	26 (14.7 %)	2 (1.9 %)	NA	<0.001
Others	42 (19.3 %)	62 (18.6 %)	1 (1.0%)	NA	49 (24.3 %)	45 (25.4 %)	21 (19.8 %)	NA	0.215
BCLC-stage*									
0-A	77 (39.1 %)	NA	NA	NA	98 (59.8 %)	NA	NA	NA	<0.001
B	44 (22.3 %)	NA	NA	NA	39 (23.8 %)	NA	NA	NA	0.765
C-D	51 (25.9 %)	NA	NA	NA	24 (14.6 %)	NA	NA	NA	0.009
Unknown	25 (12.7%)	NA	NA	NA	3 (1.8%)	NA	NA	NA	NA
*TLL1,* n (%)									
AA	177 (81.2%)	250 (75.0 %)	72 (72.7 %)	34 (64.2 %)	155 (76.7 %)	127 (71.8%)	87 (82.1 %)	5 (83.3 %)	0.262
AT	38 (17.4%)	76 (22.8 %)	26 (26.3 %)	18 (34.0 %)	32 (15.8 %)	39 (22.0 %)	17 (16.0 %)	1 (16.7 %)	0.662
TT	3 (1.4%)	7 (2.1 %)	1 (1.0%)	1 (1.9%)	15 (7.4%)	11 (6.2 %)	2 (1.9%)	0	0.003
AT/TT	41 (18.8%)	83 (25.0 %)	27 (27.3 %)	19 (35.8 %)	47 (23.3 %)	50 (28.2 %)	19 (17.9 %)	1 (16.7 %)	0.262

ALD, alcoholic liver disease; HBV, hepatitis B virus; HCV, hepatitis C virus; HCC, hepatocellular carcinoma; MASLD, metabolic dysfunction-associated steatotic liver disease; TLL1, Tolloid like 1; P-value, the comparison of variables among HCC patients from Latin America and Europe; NA, not available;

* only cirrhotic HCC patients have BCLC stage.

**Table 2:** *TLLI* rs17047200 in the development of HCC compared to cirrhosis in Latin American and European cohort

Cohort	*TLLI* genotype	Crude OR (95 % CI)	p-value	Adjusted OR (95 % CI)	p-value

Latin American	AA (reference)	-	-	-	-
	AT	0.706 (0.457–1.090)	0.117	0.699 (0.449–1.088)	0.112
	TT	0.605 (0.154–2.373)	0.471	0.694 (0.170–2.828)	0.610
	AT+TT	0.698 (0.458–1.063)	0.094	0.699 (0.456–1.072)	0.101
European	AA (reference)	-	-	-	-
	AT	0.672 (0.398–1.134)	0.137	0.598 (0.339–1.056)	0.076
	TT	1.117 (0.496–2.518)	0.789	1.244 (0.514–3.008)	0.628
	AT+TT	0.770 (0.485–1.223)	0.268	0.736 (0.447–1.211)	0.228

Note: adjusted by age, gender, and etiology (MASLD/ALD). ALD, alcoholic liver disease; HBV, hepatitis B virus; HCV, hepatitis C virus; HCC, hepatocellular carcinoma; MASLD, metabolic dysfunction-associated steatotic liver disease; TLL1, Tolloid like 1.

**Table 3:** *TLLI* rs17047200 in the development of HCC compared to cirrhosis with respective etiology in Latin American and European cohort

	Latin American				European			
	OR (95 % CI) [Crude]	p-value	OR(95 % CI) [Adjusted]	p-value	OR(95 % CI) [Crude]	p-value	OR(95 % CI) [Adjusted]	p-value

Viral (HBV+HCV)								
AT	0.321 (0.078–1.319)	0.115	0.321 (0.073–1.417)	0.134	0.612 (0.216–1.732)	0.355	0.467 (0.158–1.380)	0.168
TT	0.963 (0.057–16.214)	0.979	0.886 (0.040–19.436)	0.939	0.765 (0.133–4.407)	0.764	0.712 (0.119–4.249)	0.710
AT+TT	0.385 (0.107–1.383)	0.144	0.414 (0.110–1.560)	0.193	0.644 (0.253–1.636)	0.355	0.535 (0.204–1.405)	0.204
MASLD								
AT	0.678 (0.365–1.258)	0.218	0.698 (0.373–1.306)	0.261	0.900 (0.235–3.452)	0.878	0.589 (0.132–2.639)	0.489
TT*	NA	NA	NA	NA	1.500 (0.327–6.878)	0.602	1.945 (0.359–10.527)	0.440
AT+TT	0.630 (0.341–1.164)	0.140	0.646 (0.347–1.202)	0.168	1.125 (0.388–3.264)	0.828	1.056 (0.336–3.321)	0.926
ALD								
AT	0.602 (0.232–1.562)	0.297	0.603 (0.226–1.609)	0.312	0.233 (0.080–0.675)	**0.007**	0.218 (0.071–0.668)	**0.008**
TT	1.355 (0.082–22.513)	0.832	1.299 (0.077–21.809)	0.856	1.034 (0.108–9.950)	0.977	0.634 (0.061–6.572)	0.703
AT+TT	0.642 (0.256–1.609)	0.344	0.642 (0.249–1.655)	0.359	0.306 (0.114–0.817)	**0.018**	0.273 (0.096–0.773)	**0.015**

Note: adjusted by gender and age. Reference allele of *TLL1* is AA.

* In Latin American cohort, none of MASLD patients with HCC had *TLL1* genotype TT. ALD, alcoholic liver disease; HBV, hepatitis B virus; HCV, hepatitis C virus; HCC, hepatocellular carcinoma; MASLD, metabolic dysfunction-associated steatotic liver disease; TLL1, Tolloid like 1.

## Abbreviations:

ALD: alcoholic liver disease
HBV: hepatitis B virus
HCV: hepatitis C virus
HCC: hepatocellular carcinoma
MASLD: with metabolic dysfunction-associated steatotic liver disease
OR: odds ratio
SNPs: single nucleotide polymorphisms
TLL1: Tolloid like 1
